# Multimodal Analgesia Bundle and Postoperative Opioid Use Among Patients Undergoing Colorectal Surgery

**DOI:** 10.1001/jamanetworkopen.2023.32408

**Published:** 2023-09-06

**Authors:** Claes Gedda, Jonas Nygren, Anna Garpenbeck, Linda Hoffström, Anders Thorell, Mattias Soop

**Affiliations:** 1 Department of Surgery and Anesthesiology, Ersta Hospital, Stockholm, Sweden; 2 Karolinska Institutet at Danderyd Hospital, Stockholm, Sweden; 3 Department of Inflammatory Bowel Disease and Intestinal Failure Surgery, Karolinska University Hospital, Stockholm, Sweden

## Abstract

**Importance:**

A key objective in contemporary surgery is to reduce or eliminate the usage of opioids to minimize gastrointestinal adverse effects, fatigue, and long-term opioid dependency.

**Objectives:**

To evaluate the association of the implementation of a care bundle of 3 opioid-sparing interventions with the amount of opioids consumed postoperatively among patients undergoing major abdominal surgery and to examine the respective associations of the 3 components.

**Design, Setting, and Participants:**

This retrospective cohort study was performed at Ersta Hospital, an elective teaching hospital in Stockholm, Sweden. All patients undergoing major colorectal surgery between January 1, 2016, through December 31, 2019, were included. Data analysis was conducted from February 1, 2020, to May 30, 2022.

**Exposures:**

A care bundle consisting of an individualized opioid regimen, regular gabapentinoids, and clonidine as a rescue analgesic was gradually introduced early in the study period.

**Main Outcomes and Measures:**

Amount of in-hospital administered intravenous and oral opioids on the day of surgery and the first 5 postoperative days (morphine milligram equivalents [MME]). The association between exposure and outcome was examined using multivariable linear regression.

**Results:**

Overall, 842 patients had major colorectal surgery in the study period (mean [SD] age, 64.6 [15.5] years; 421 [50%] men). Median (range) opioid usage decreased from 75 (0-796) MME in 2016 to 22 (0-362) MME in 2019 (*P* < .001), and the proportion of patients receiving 45 MME or less increased from 35% to 66% (*P* < .001). On multivariable analysis (F_5, 836_ = 57.5; *P* < .001), an individualized opioid strategy (β = −11.6; SE = 3.8; *P* = .003), the use of gabapentin (β = −39.1; SE = 4.5; *P* < .001), and increasing age (β = −1.0; SE = 0.11; *P* < .001) were associated with less opioid consumption, while the use of clonidine was associated with more opioid intake (β = 11.6; SE = 3.6; *P* = .001).

**Conclusions and Relevance:**

In this cohort study of 842 patients undergoing colorectal surgery, a care bundle consisting of an individualized opioid regimen, regular gabapentin, and clonidine as a rescue analgesic was found to be associated with a significant decrease in opioids consumed postoperatively. Regular gabapentin and an individualized opioid regimen were particularly strongly associated with this decrease and should be further evaluated as components of multimodal, opioid-free postoperative analgesia.

## Key Points

**Question:**

Is a care bundle consisting of an individualized opioid regimen, regular gabapentinoids, and clonidine associated with postoperative opioid consumption among patients after major colorectal surgery?

**Findings:**

In this cohort study of 842 patients, gabapentin and an individualized opioid regimen were associated with a significant decrease of opioid consumption after major colorectal surgery so that the proportion of patients receiving low or no opioids increased from 35% to 66%.

**Meaning:**

These findings suggest that an individualized opioid regimen and the regular use of gabapentin may minimize and, in many cases, eliminate the need for opioid analgesia after colorectal surgery.

## Teaser

This cohort study evaluates the association between the implementation of a care bundle with 3 opioid-sparing interventions and the amount of opioids consumed postoperatively among patients undergoing major abdominal surgery and examines the respective associations of each of the components.

## Introduction

The opioid epidemic affects large parts of the world, causing an estimated 350 000 deaths annually worldwide.^1^ In 2019, 70 630 people died from an opioid overdose in the United States, and in 28% of those, medically prescribed opioids were involved.^2^ The Organization for Economic Co-operation and Development (OECD) has reported that the opioid epidemic now also affects Europe, where Sweden has the second-highest incidence of opioid-related deaths.^3^

In the United States, surgeons issued approximately 30 million opioid prescriptions in 2012, constituting about 10% of all prescribed opioids.^4^ Prescribed opioids are consumed not only by the patient but often also by relatives and friends^5^; indeed, the main source of abused prescription opioids is family and friends.^6^

In addition to addiction, opioids have adverse effects that impair recovery after surgery, such as impaired gastrointestinal motility and impaired mobilization.^7,8,9^ Enhanced recovery after surgery (ERAS) protocols use a range of opioid-sparing strategies. Multimodal analgesia is an approach in which several analgesics are combined to maximize analgesia while minimizing their adverse effects.^10^ Regional analgesic techniques, such as epidurals, spinal blocks, transverse abdominis plane blocks, and rectus blocks, are widely used.^11^

Additional methods have been proposed to reduce the need for opioids in postoperative pain control. Those include individualized opioid dosages,^12^ gabapentinoids as a baseline analgesic,^13^ and clonidine as a rescue analgesic.^14,15^ However, the opioid-sparing effects of these methods have not yet been well evaluated in major abdominal surgery.

In this retrospective cohort study of prospectively collected perioperative data, we evaluated the association of implementing a care bundle consisting of an individualized opioid regimen, regular gabapentinoids, and clonidine as a rescue analgesic and the amount of opioids administered postoperatively among patients undergoing major abdominal surgery. Additionally, the associations between each intervention and opioid administration were analyzed.

## Methods

### Study Design

This is a single-center, retrospective cohort study that was approved by the Swedish Ethical Review Authority, which did not require informed consent due to the retrospective nature and size of the study. This study is reported according to the Strengthening the Reporting of Observational Studies in Epidemiology (STROBE) protocol.

### Setting

Ersta Hospital is an elective 65-bed university-affiliated hospital in Stockholm, Sweden. It specializes in high-volume care for gastrointestinal diseases.

### Participants

All patients undergoing colorectal surgery via laparoscopy or laparotomy at the study site between the January 1, 2016, and the December 31, 2019, were included. Patients undergoing surgery by parastomal incision alone were not included. No other exclusion criteria were applied. To ensure the integrity of the individual study participant, all data were deidentified prior to analysis, and all analyses were performed on the group level.

### Interventions

During the 4-year study period, a multimodal care bundle was implemented aiming to reduce reliance on opioid analgesia in the postoperative period. The care bundle consisted of 3 interventions: an individualized opioid regimen, regular gabapentinoids, and clonidine as a rescue analgesic.

The individualized opioid regimen involved replacing a standard order set consisting of oral oxycodone at 10 mg and naloxone at 5 mg twice per day, with a new order set consisting of oral oxycodone at 5 mg on demand, given until satisfactory pain relief (pain score ≤4 on a scale from 0 to 10). The change of the order set was performed through an alteration of the electronic prescription system on July 1, 2017, preceded by educational efforts.

Regular gabapentinoids were implemented as a standard order set of oral gabapentin 300 mg twice on the day of surgery followed by 300 mg 3 times daily from day 1 until 7 to 10 days after surgery. A lower dose was used in patients aged 80 years or older and in patients with a reduced renal clearance (estimated glomerular filtration rate of less than 50 mL/min/1.73 m^2^). To avoid possible adverse events caused by gabapentinoids individually or when combined with opioids, the dosage chosen is the lowest adult dose recommended by the on-label treatment recommendations issued by the US Food and Drug Administration.^16^

Clonidine rescue analgesia was implemented as a standard order set of a single dose of 75 µg administered intravenously on-demand for rescue pain relief, instead of opioids being used as initial rescue pain relief. Clonidine was not given in the presence of bradycardia (heart rate <50 beats/min) or hypotension (mean arterial pressure <65 mmHg).

Gabapentinoids and clonidine were introduced gradually in the first year, supported by educational efforts and alterations in the electronic prescription system. Compliance was measured parallel to the implementation and further educational resources were applied as needed. All adverse effects related to the interventions were recorded.

### Ancillary Management

All patients were cared for in an established ERAS perioperative care pathway, optimizing a range of aspects of perioperative care, such as fluid therapy, nutrition, and mobilization.^17^ Regarding perioperative pain control, patients undergoing open surgery received epidural analgesia for 2 to 4 days, while patients having laparoscopic procedures received spinal analgesia. All patients received oral acetaminophen at 1 g 4 times per day unless contraindicated. Oral opioids were started, as detailed previously, when the effects of the spinal analgesia had abated or the epidural analgesia was terminated.

On discharge, patients were given acetaminophen at 1 g 4 times daily and gabapentin at 300 mg 3 times daily for 2 weeks after surgery as well as short-acting oxycodone at 5 mg for rescue analgesia. The prescription for short-acting oxycodone was limited to the smallest package available, 14 doses.

### Variables and Data Sources

#### Outcomes

The primary outcome measure was the cumulative amount of opioids administered orally or intravenously during the day of surgery and the first 5 postoperative days. Different opioids were converted to morphine milligram equivalents (MME), as defined by the US Centers for Disease Control and Prevention.^18^ Intrathecal and epidural opioids were not included in the cumulative amount, as the systemic availability and adverse effects of such administration are likely to be minor.^19,20,21,22,23^ Secondary outcome measures were the proportion of patients receiving no opioids during the day of surgery and the 5 days following surgery, as well as the proportion receiving low-dose opioids defined as 45 MME or less, corresponding to 3 doses of oral oxycodone at 10 mg over the 6-day study period.

#### Independent Variables

For each patient, the 3 interventions were assessed as present or absent as follows. The individualized opioid regimen was present in patients who received this order set. Patients were recorded as treated with regular gabapentinoids if they were prescribed this order set and received at least 6 doses during the study period. Clonidine use was recorded in patients who received this order set and at least 1 dose during the study period. The administration was confirmed by the nursing staff, following routine clinical practice. The following data were also recorded: sex, age (years), and body mass index (calculated as weight in kilograms divided by height in meters squared) at the time of surgery, smoking status, primary diagnosis (malignant disease, inflammatory bowel disease, diverticular disease, and other benign disease), surgical approach (laparoscopic or open surgery including converted operations), the magnitude of surgery (abdominal or abdominopelvic), duration of surgery (minutes), type of regional analgesia (epidural, spinal, or none), American Society of Anesthesiologists (ASA) risk class (I-V), compliance to the components of the ERAS protocol (percentage), and the highest Clavien-Dindo^24^ complication grade within 30 days of surgery (0-V).

#### Data Sources

The cohort was studied longitudinally. Data on demographic characteristics and perioperative variables were collected from an international perioperative audit database, the Enhanced Recovery After Surgery Interactive Audit System (EIAS)^25^ and from electronic medical records.

### Statistical Analysis

Nominal and ordinal data are presented as proportions, and continuous data are presented as their mean with SD or median with range, as appropriate. Differences between groups in nominal or ordinal data were assessed by the χ^2^ test. Continuous data were assessed by Student *t* test or Wilcoxon rank sum test, as appropriate. *P* values were 2-tailed, and results were deemed significant at *P* < .05. A robust linear regression was used to compensate for outliers and nonuniform variance using Stata version 13 (StataCorp).

Analysis of data was performed between February 1, 2020, and the May 30, 2022. All putative factors associated with postoperative opioid consumption were analyzed by univariable and multivariable linear regression. Backward stepwise selection was performed to identify variables included in the final model.

## Results

### Patient Characteristics

A total of 842 patients had colorectal surgery between January 1, 2016, and December 31, 2019 (mean [SD] age, 64.6 [15.5] years; 421 [50%] men) (Table 1). None were lost to follow-up. The mean (SD) duration of surgery was 256 (114) minutes. Mean (SD) compliance with the ERAS pathway was 74% (12), and the median (range) postoperative length of stay was 5 (1-49) nights; 274 patients (33%) were discharged before the end of the observation time.

**Table 1.** Clinical Data for 842 Patients Undergoing Major Colorectal Surgery, 2016-2019

Characteristic	Patients, No. (%)
Age, mean (SD), y	64.6 (15.5)
Sex	
Male	421 (50)
Female	421 (50)
BMI, median (range)	25.3 (15.1-41.0)
ASA score	
I	104 (12)
II	545 (65)
III	192 (23)
IV	1 (0.12)
Diagnosis	
Malignant disease	509 (60)
Inflammatory bowel disease	130 (15)
Diverticular disease	88 (10)
Other benign disease	115 (14)
Magnitude of surgery	
Abdominal	600 (71)
Abdomino-pelvic	242 (29)
Regional analgesia	
Epidural	604 (72)
Spinal	231 (27)
No block	7 (1)
Surgical approach	
Minimally invasive surgery	608 (72)
Open surgery	234 (28)
Individualized opioid treatment	
Yes	534 (63)
No	308 (37)
Gabapentin	
Treated	661 (79)
Untreated	181 (21)
Clonidine	
Treated	246 (29)
Untreated	596 (71)

Abbreviations: ASA, American Society of Anesthesiologists; BMI, body mass index (calculated as weight in kilograms divided by height in meters squared).

### Implementation of the Care Bundle

**Figure 1.**
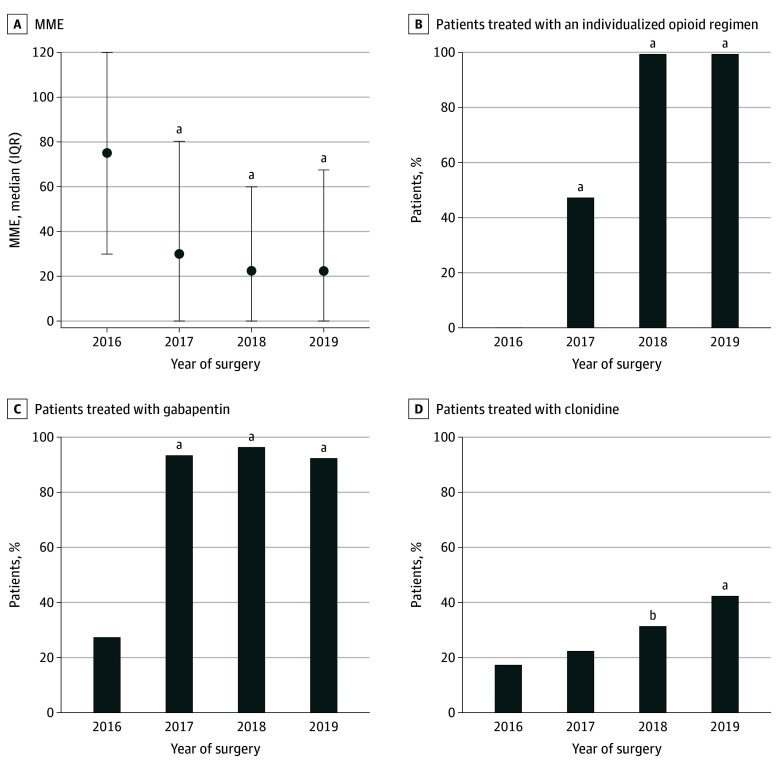
Total Morphine Milligram Equivalents (MME) on the Day of Surgery and Subsequent 5 Days and Proportions of Patients Receiving Each Component of the Multimodal Bundle A, For 842 patients undergoing major colorectal surgery, dots indicate medians, and whiskers indicate IQR. *P* values indicate the result of the *t* test with the value of 2016 as the baseline. ^a^*P* < .001. ^b^*P* < .05.

The use of all 3 elements of the care bundle increased during the study period (Figure 1). From 2016 to 2019, the individualized opioids increased from 0% (0 of 206) to 100% (207 of 207) (χ^2^ test not applicable), regular gabapentin from 28% (58 of 206) to 93% (193 of 207) (413 patients; χ^2^ = 183.4; *P* < .001), and clonidine rescue from 18% (37 of 206) to 43% (89 of 207) (413 patients; χ^2^ = 30.5; *P* < .001). The use of clonidine as rescue analgesia increased in 2018, while the use of the other 2 interventions increased already in 2017 (Figure 1). In 15 patients (2.3%) who received regular gabapentin, the drug was discontinued due to a suspected adverse event: sedation (6 patients), confusion (5 patients), visual disturbances (3 patients), and unsteadiness (5 patients). Among patients in whom the drug was discontinued, the mean (SD) age was 74.0 (14.3) years and 11 (73%) were women.

### Opioid Usage

**Figure 2.**
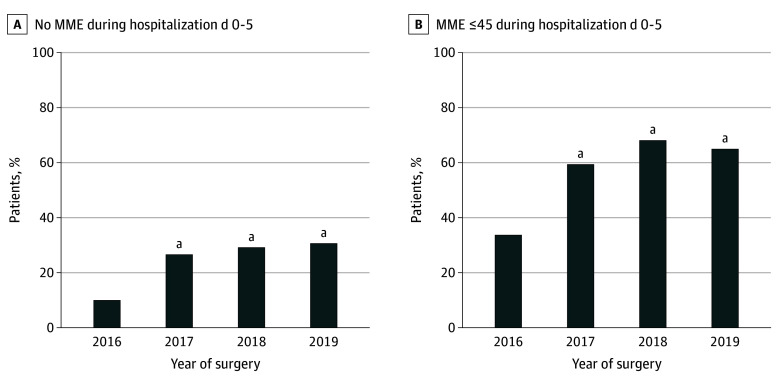
Proportion of Patients Who Received No Systemic Opioids and 45 Morphine Milligram Equivalent (MME) or Less Systemic Opioids *P* values indicate the result of the *t* test with the value of 2016 as the baseline. ^a^*P* < .001.

Median (range) opioid use decreased in parallel to the implementation of the care bundle, from 75 (0-796) MME in 2016 to 22 (0-362) MME in 2019 (*z* = 7.125; *P* < .001) (Figure 1). The proportion of patients who did not consume any opioids in-hospital at all during the 6-day study period increased gradually over the years of the study, from 11% (22 of 206) in 2016 to 31% (65 of 207) in 2019 (413 patients; χ^2^ = 12.1; *P* < .001) (Figure 2A). Similarly, patients receiving low doses (≤45 MME) during the 6 days increased from 35% (71 of 206) to 66% (136 of 207) during the study (413 patients; χ^2^ = 24.5; *P* < .001) (Figure 2B).

### Factors Associated With Opioid Usage

On univariable analysis (Table 2), the use of an individualized opioid regimen, regular gabapentin, increased age, spinal analgesia, and increased compliance to the ERAS protocol were associated with less opioid usage. Meanwhile, clonidine use, smoking, and a diagnosis of inflammatory bowel disease were associated with increased usage.

**Table 2.** Univariable Linear Regression Analysis for Accumulated Milligram Morphine Equivalents During Days 0 to 5 After Major Colorectal Surgery in 842 Patients

Factor	Coefficient (SE)	*t* Ratio	*P* value
Minimally invasive surgery (yes vs no)	6.01 (3.9)	1.54	.12
Individualized opioid regimen (yes vs no)	−24.29 (3.49)	−6.96	<.001
Gabapentin (yes vs no)	−38.06 (4)	−9.51	<.001
Clonidine (yes vs no)	15.08 (3.84)	3.92	<.001
Sex (female vs male)	4.74 (3.53)	1.34	.18
Age, y	−0.92 (0.11)	−8.41	<.001
BMI	−0.30 (0.39)	−0.76	.45
ASA group (I-II vs III-IV)	4.65 (4.2)	1.11	.27
Smoker (yes vs no)	17.25 (7.72)	2.24	.03
Diagnosis (IBD vs other)	29.71 (4.74)	6.27	<.001
Magnitude of surgery (abdomino-pelvic vs abdominal)	−5.81 (3.88)	−1.5	.14
Regional analgesia (spinal vs epidural)	−10.36 (3.87)	−2.67	.008
Compliance to ERAS protocol components (%)	−0.72 (0.14)	−5.10	<.001
Complications (yes vs no)	−4.99 (4.58)	−1.09	.28

Abbreviations: ASA, American Society of Anesthesiologists; BMI, body mass index (calculated as weight in kilograms divided by height in meters squared); ERAS, enhanced recovery after surgery; IBD, inflammatory bowel disease.

In the final multivariable model (F_5, 836_ = 57.5; *P* < .001) (Table 3), an individualized opioid strategy (β = −11.6; SE = 3.8; *P* = .003), regular gabapentin (β = −39.1; SE = 4.5; *P* < .001), and increased age (β = −1.0; SE = 0.11; *P* < .001) were associated with a decrease of opioid consumption. The use of clonidine remained associated with increased opioid intake (β = 11.6; SE = 3.6; *P* = .001).

**Table 3.** Multivariable Linear Regression Analysis for Accumulated Milligram Morphine Equivalents During Days 0 to 5 After Major Colorectal Surgery in 842 Patients, After Backward Selection of Covariates^a^

Factor	Coefficient (SE)	*t* Ratio	*P* value
Individualized opioid regimen (yes vs no)	−11.57 (3.83)	−3.02	.003
Gabapentin (yes vs no)	−39.14 (4.52)	−8.66	<.001
Clonidine (yes vs no)	11.63 (3.61)	3.23	.001
Age, y	−1.02 (0.11)	−9.62	<.001

^a^ There were 842 observations (F_5, 836_ = 57.49; *P* < .001).

## Discussion

In this large, consecutive cohort of patients undergoing major colorectal surgery, we found that implementation of a multimodal care bundle was associated with a significant decrease in postoperative opioid consumption. In the final year of the study, the proportion of patients receiving less than 45 MME orally or parenterally over the first 6 days after surgery was 66%, corresponding to 3 doses of oxycodone at 10 mg. Furthermore, 31% received no opioids at all during the same period. These data demonstrate that the goal of nearly opioid-free major surgery is achievable in routine clinical practice, using alternative modalities to manage postoperative pain.

As the global opioid crisis kills more people each year, it is paramount that physicians prescribe as few doses of opioids as possible. In a study by Brummett et al^26^ it was reported that 10% of patients undergoing colectomy had developed new persistent opioid use after surgery.

In this study, the use of regular gabapentin was most strongly associated with a decrease in opioid consumption. Only 2% of study participants discontinued gabapentin due to a suspected adverse reaction. The adverse reactions were all mild and fully reversible.

The results of this study contradict findings in the most recent meta-analysis on gabapentinoids in postoperative analgesia, which concluded that gabapentinoids provide clinically irrelevant analgesia and are associated with too many serious adverse effects to be considered effective.^27^ Conversely, in our study, the association between gabapentin and a decrease in opioid consumption was significant and did not come at the cost of serious adverse reactions. We believe that dosing and the timing of the doses can be a part of an explanation. In our study, we administered 300 mg of gabapentin 3 times daily, a low dose in the normal range of prescription. The Food and Drug Administration has issued a warning regarding the risk of respiratory depression and other serious adverse events when gabapentinoids are combined with central nervous system depressants, such as opioids.^28^ Most participants in our study consumed no or small amounts of opioids, which may explain the absence of respiratory depression.

Importantly, the present study differs from previous literature on gabapentinoids in that the drug was administered regularly after colorectal surgery, rather than given as a premedication before surgery. The 7 studies on colorectal surgery included in the recent meta-analysis only provided the drug preoperatively.^27^ Three of the 81 included studies on abdominal surgery administered gabapentinoids longer than 1 day postoperatively: all 3 concerned hysterectomy. In a study by Singla et al,^29^ pregabalin at 150 mg was found to reduce opioid requirements after hysterectomy, although pregabalin at 300 mg was not. Fassoulaki et al have published 2 studies on this topic^30,31^: in one, regular pregabalin was found to decrease opioid requirements during the first 48 hours after surgery; in the second, regular gabapentin showed no association with acute postoperative opioid consumption, although pain scores were lower 3 months after surgery in the gabapentin group.

Several studies emphasize a preemptive analgesic effect,^32,33^ although the mechanism is poorly understood. However, such an effect does not exclude the possibility that regularly administered gabapentinoids could also prove beneficial.

An individualized opioid protocol was also associated with decreased consumption of postoperative opioids. We hypothesize that the main reason for this finding is a previous unnecessary overdispensation of postoperative opioids. There is likely to be a mismatch between a standard opioid order set and the true opioid requirement of the individual patient. Patients undergo a wide range of surgical procedures, from a 90-minute right hemicolectomy to a 480-minute abdominoperineal proctectomy. Furthermore, patients vary widely regarding ASA class, age, and weight. For some patients, opioids are a necessary part of the multimodal jigsaw puzzle, and for some they are less necessary. The finding of highly variable opioid requirements each year of the present study emphasizes this individual need for analgesia and confirms previous findings that a 1-size-fits-all model for postoperative analgesia is associated with both overadministration and underadministration of opioids, an increase in pain and adverse events,^34^ a longer stay in hospital, and a higher cost of care.^35^

The use of clonidine as rescue analgesia was associated with an increase in opioid consumption. This finding may indicate that patients who required clonidine also required additional doses of opioids, the second-line rescue analgesia when clonidine was used. In a systematic review by Blaudszun et al,^14^ clonidine was found to have a discreet mean opioid-sparing effect of 4.1 mg when used regularly after surgery, an outcome we failed to reproduce in this study.^14^ In the setting of our study, clonidine was used per request, which leads us to believe that it rather became a marker for an increase in pain.

Contrary to previously published studies, the increased use of minimally invasive surgery was not associated with opioid consumption.^36,37^ However, one must consider that in the context of our study, the surgical approach dictated which type of axial block was used. Patients undergoing open surgery frequently received an epidural (94%), whereas those operated laparoscopically often were administered spinal analgesia (63%). Epidural analgesia is commonly used over several days, while spinal analgesia remains for approximately 16 hours. This may partly explain this finding.

Opioid-free postoperative care has recently been suggested as providing better outcomes after colorectal surgery. In a study from 2019, Keller et al^38^ analyzed 50 098 colorectal cases with respect to the association between opioid consumption and total cost, length of stay, and the number of readmissions. The authors of this study found that 2919 of 50 098 cases (6%) were considered opioid-free postoperatively, and this group had significantly lower total costs, lower length of stay, and fewer readmissions. In the present study, 31% of patients consumed no opioids at all during the last year of observations when the care bundle was fully implemented.

### Limitations

We acknowledge that there are several limitations to this study. Implementation of the individual components of the care bundle was unevenly distributed over time as a result of the complexity of changing clinical practice, prohibiting a direct comparison of data before vs after implementation. The retrospective design inherently limits conclusions regarding causality. Furthermore, unmeasured changes in attitudes toward opioid usage are likely to have occurred during the study period, which also may have contributed to a decrease in usage. Opioid usage after discharge was not measured, potentially underestimating the amount of opioids consumed. However, opioid doses on discharge were low, and only a minority of patients were discharged before the end of the 6-day study period (274 [33%]). However, we regard the size of the study and the inclusion of all consecutive patients undergoing operations during the 4-year observation period to be relevant strengths of this study, limiting inclusion bias.

## Conclusions

In this cohort study, a care bundle including an individualized opioid regimen, regular gabapentin, and clonidine as a rescue analgesic was associated with a significant decrease in the amount of opioids consumed after major colorectal surgery. Gabapentin and an individualized opioid regimen were strongly and independently associated with this decrease and should be further evaluated as components of multimodal, opioid-free postoperative analgesia.

## Notes

Supplement.
Data Sharing Statement
