# Error in Methods Section

**DOI:** 10.1001/jamanetworkopen.2026.2546

**Published:** 2026-02-26

In the Original Investigation titled “Multimodal Analgesia Bundle and Postoperative Opioid Use Among Patients Undergoing Colorectal Surgery,”^1^ published September 6, 2023, there was an error in the Methods section. In the fourth paragraph of the “Interventions” subsection, the dose for clonidine should have read 75 μg. This article has been corrected.^1^
