# Supplementary material for: Multimodal Analgesia Bundle and Postoperative Opioid Use Among Patients Undergoing Colorectal Surgery
Source: JAMA Netw Open. 2023 Sep 6;6(9):e2332408. doi: 10.1001/jamanetworkopen.2023.32408 (PMC10483316; doi:10.1001/jamanetworkopen.2023.32408)
Supplement: Supplement. — Data Sharing Statement [file jamanetwopen-e2332408-s001.pdf]

Data Sharing Statement

Gedda. Multimodal Analgesia Bundle and Postoperative Opioid Use Among Patients Undergoing Colorectal Surgery. *JAMA Netw Open*. Published September 06, 2023. doi:10.1001/jamanetworkopen.2023.32408

Data
Data available: No
